# Comparative effectiveness and toxicity of radiotherapy regimens in limited stage small cell lung cancer: A network meta‐analysis

**DOI:** 10.1002/cam4.4774

**Published:** 2022-04-25

**Authors:** Jiupeng Zhou, Hui Guo, Yongfeng Zhang, Heng Liu, Quanli Dou

**Affiliations:** ^1^ Xi'an Chest Hospital Xi'an China; ^2^ The First Affiliated Hospital of Xi'an Jiaotong University Xi'an China

**Keywords:** effectiveness, limited‐stage small cell lung cancer, network meta‐analysis, radiotherapy regimens, toxicity

## Abstract

**Purpose:**

The aim of this Network Meta‐analysis was to compare the current radiotherapy regimens of limited‐stage small cell lung cancer (LS‐SCLC), in terms of overall survival (OS), progression‐free survival (PFS), and the incidence of acute radioactive esophagitis and radioactive pneumonia.

**Methods:**

PubMed, Embase, Web of Science, and the Cochrane Library were comprehensively searched until January 2022. The studies were included, comparing radiotherapy regimens in LS‐SCLC patients. We compared hypofractionated radiotherapy (HypoTRT), hyperfractionated radiotherapy (HyperTRT), and conventionally fractionated radiotherapy (ConvTRT1(<60 Gy), ConvTRT2(≥60 Gy)).

**Results:**

There was similar efficacy among the contemporary radiotherapy regimens for PFS of LS‐SCLC. HypoTRT and HyperTRT significantly improved the OS of LS‐SCLC compared with ConvTRT1 (<60 Gy), while not improving the OS of LS‐SCLC compared with ConvTRT2 (≥60 Gy). There was no significant difference between HypoTRT and HyperTRT, between ConvTRT1(<60 Gy) and ConvTRT2(≥60 Gy), respectively. HyperTRT developed the highest odds of acute radioactive esophagitis compared to ConvTRT1(<60 Gy) and ConvTRT2(≥60 Gy). There was no significant difference in the incidence of acute radioactive esophagitis between HypoTRT and HyperTRT, ConvTRT1(<60 Gy), ConvTRT2(≥60 Gy), respectively and between ConvTRT1 and ConvTRT2. There was no statistically significant difference among radiotherapy regimens for the incidence of acute radioactive pneumonia.

**Conclusion:**

The current radiotherapy regimens are similar in efficacy and toxicity for LS‐SCLC, except for ConvTRT1(<60 Gy). Given the lower costs and convenient logistics management of HypoTRT comparatively, it is an acceptable alternative for LS‐SCLC.

## Graphical abstract

This network meta‐analysis aimed to compare the value ofthe current radiotherapy regimens as estimated by PFS and OS for HypoTRT, HyperTRT, and ConvTRT, aswell as to compare the adverse events and relative toxicity of radiotherapy regimens.

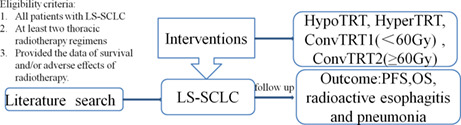

## 1 INTRODUCTION

It is estimated that lung cancer ranks second among common tumors and is the main cause of tumor death. In 2020, there were more than 2 million newly diagnosed lung cancers and 1.8 million deaths from lung cancer, accounting for approximately one tenth (11.4%) of the tumors diagnosed and one fifth (18%) of the deaths.^1^ In the United States, there are about 30,000 patients with small cell lung cancer (SCLC) every year, with a 5‐year overall survival rate of 6%.^2^ This deadly neuroendocrine tumor with a short doubling time and early metastasis is difficult to treat for oncologists.^3^ For limited‐stage small cell lung cancer(LS‐SCLC), the standard treatment is concurrent chemoradiotherapy at present.^4^, ^5^, ^6^ However, the optimal dose and fractionations of radiotherapy are still controversial.

Hyper/ConvTRT have been recommended by the NCCN on the basis of clinical efficacy in separate clinical trials,^7^, ^8^, ^9^, ^10^, ^11^ but it is not clear which treatment is better. Turrisi et al^12^ and Faivre‐Finn C et al^7^ reported that the hyperfractionated, twice‐daily radiotherapy (BID) demonstrated superior survival when compared to the conventionally fractionated thoracic radiotherapy. Yet, this result was not widely accepted due to inconvenient operation and potential severe esophagitis and granulocytopenia.^13^ The latest two prospective studies reported that hypofractionated radiotherapy (HypoTRT) seemed to contribute to improve OS than HyperTRT.^14^, ^15^ In 2020, two meta‐analyses revealed that twice‐daily thoracic radiotherapy appeared to be better than once‐daily for LS‐SCLC with better antitumor effects (OS) and similar adverse effects. However, neither did the two studies strictly distinguish HypoTRT and ConvTRT in once‐daily thoracic radiotherapy group, nor did they consider the effect of total doses in ConvTRT group.

So, the primary objective of this network meta‐analysis was to compare the value of the current radiotherapy regimens as estimated by PFS and OS for HypoTRT, HyperTRT, and ConvTRT. The secondary objective was to compare the adverse events and relative toxicity of radiotherapy regimens mentioned above. Network meta‐analysis can indirectly compare A and B therapies through a common comparator, such as C therapy that has been compared to A and B therapies in different studies.^16^

## 2 MATERIALS AND METHODS

### 2.1 Search strategy

PubMed, Embase, Web of Science, and the Cochrane Library were comprehensively searched for studies that evaluated radiotherapy regimens for LS‐SCLC. The search work ended in January 2022. The following search terms were used: small cell lung cancer, radiotherapy, hypofractionated, hyperfractionated, conventionally fractionated radiotherapy, twice‐daily, and once‐daily etc. The study was confined to English publications focusing on humans.

### 2.2 Inclusion and exclusion criteria

Inclusion criteria: (1) All patients were ones with LS‐SCLC; (2) the studies compared at least two thoracic radiotherapy regimens; (3) the studies provided the data of survival and/or adverse effects of radiotherapy. Exclusion criteria: Single arm studies, case reports, repeated publications, studies with unclear efficacy indicators, and without control group, conference abstracts without full‐text and registered clinical studies with incomplete key data were excluded.

### 2.3 Interventions and subjects

Interventions: HypoTRT, HyperTRT, and ConvTRT. ConvTRT was divided into ConvTRT1(<60 Gy) and ConvTRT2(≥60 Gy) on the basis of the minimum total radioactive dose in the group. Subjects: Patients with LS‐SCLC diagnosed by pathology, regardless of gender, race, and nationality.

### 2.4 Data extraction and quality assessment

The data extraction was conducted by two investigators independently. Any disagreements were discussed and reached an agreement with the participation of the third researcher. The following data were extracted: the first author, publication year, country, sample size, median age of participants, chemotherapy regimen, radiotherapy regimen, follow‐up time, adverse reactions (esophagitis and pneumonia), and survival data (PFS,OS). HR was first extracted from multivariate analysis, if not, HR from univariate analysis. Once HR was missing, HR was estimated from Kaplan–Meier curve with the method recommended by Tierney et al.^17^ and Parmar.^18^ The studies were evaluated according to NOS scale and 6 ~ 9 points were high‐quality literature. The study obtained approval from the Ethics Committee of Xi'an Chest Hospital.

### 2.5 Statistical analysis

WinBUGS 14.0 and Stata 14.0 were used for data analysis. The estimated risk ratio (RR) was used to summarize the relationship between interventions and esophagitis or pneumonia. The prognostic role of interventions was evaluated by both adjusted and unadjusted hazard ratios (HRs) and their 95% confidence intervals (CIs) of OS/PFS on behalf of survival from the primary studies. The rank probability for each treatment was estimated and illustrated visually using bar diagram. The consistency of the network was evaluated by Lumley method using Stata 14.0. Publication bias was assessed by funnel diagram.

## 3 RESULTS

### 3.1 Studies identification and characteristics of eligible studies

**FIGURE 1:**
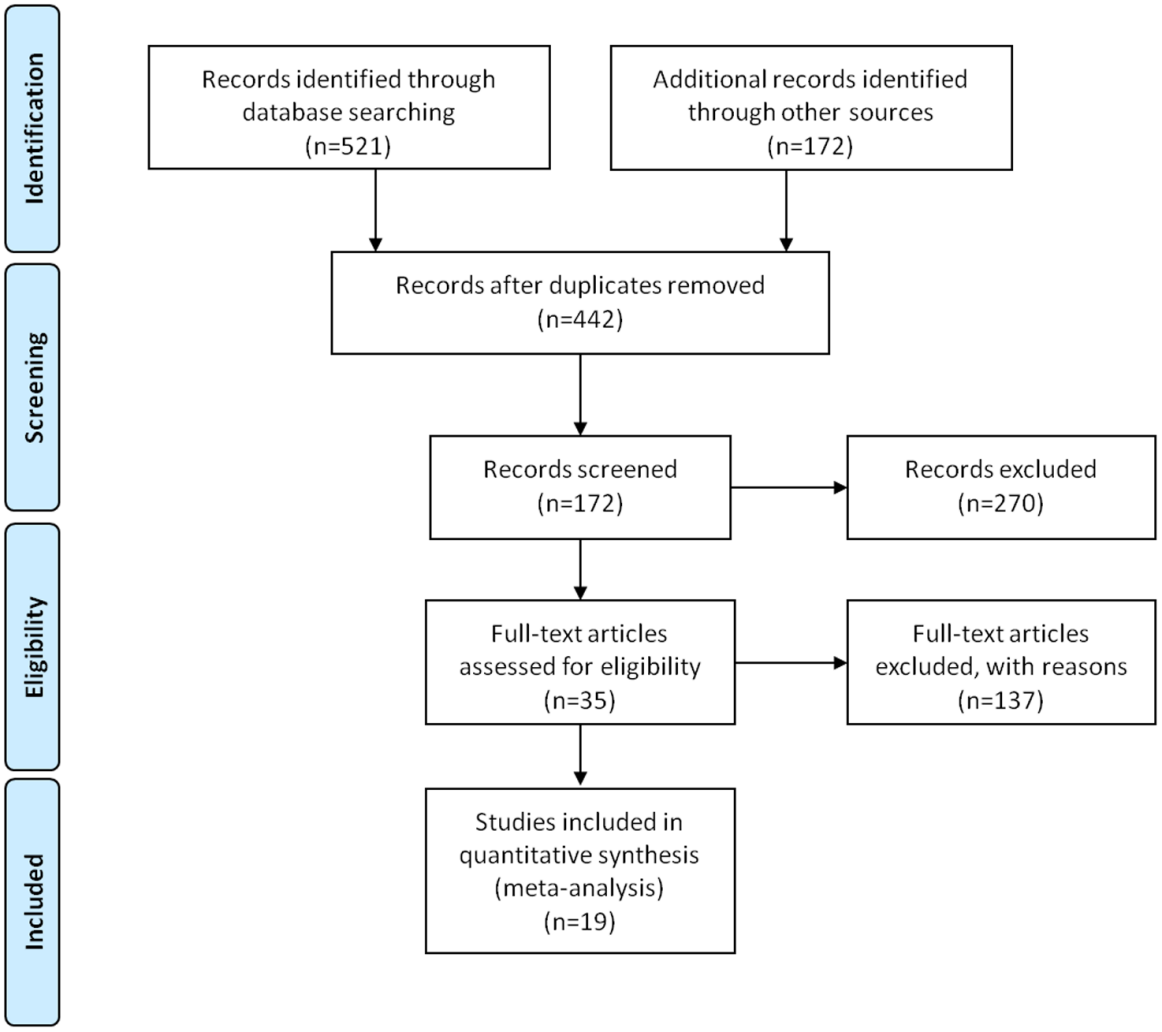
A flowchart describing the procedures of document retrieval and selection

Of the 693 potentially relevant articles, 658 articles were excluded by title and abstract in the initial screening stage, leaving only 35 for full‐text evaluation. In the rest articles, the articles were excluded those did not include extractable survival data or the regimens of interest (HyperTRT and ConvTRT were mixed into a group). Finally, 19 articles were included in this study, reporting the results of eight prospective studies^7^, ^8^, ^12^, ^14^, ^15^, ^19^, ^20^, ^21^ an 11 retrospective ones.^22^, ^23^, ^24^, ^25^, ^26^, ^27^, ^28^, ^29^, ^30^, ^31^, ^32^ (Figure 1).

Table 1 provided the characteristics of the included studies. Specific details about the total dose and fraction of each radiotherapy regimen were presented. The patients received at least one cycle of concurrent chemotherapy during radiotherapy. Our study included 28,189 patients. They came from America, Poland, Australia, Norway, China, Canada, UK, Netherlands, and Japan. PFS, OS, radioactive esophagitis, and radioactive pneumonia were reported in 14, 19, 11, and 11 studies, respectively. The scores of these all studies were ≥7 points by NOS scale.

**TABLE 1:** The basic information and data of all included studies in the meta‐analysis

Study	Country	No. of patients	Arm	Median age (years)	Median follow‐up (months)	Quality score of literature	Study design	Radioactive Esophagitis	Radioactive pneumonitis	PCI	CTregimen
Gregory M. M. (2003)	America	122	HypoTRT40 Gy/15f	63	14.8	7	Retrospective			21	CAV /CEV /EP
		92	Conv TRT 50 Gy/25f							27	
Catherine S. Bettington (2013)	Australia	38	HypoTRT 40 Gy/15f	66.5	≥12	8	Retrospective			19	EP /EC
		41	HyperTRT45 Gy/30f	61						28	
Bjørn H. Grønberg (2015)	Norway	84	HypoTRT 42 Gy/15f	63	81	7	Prospective	26	5	69	EP /EC
		73	HyperTRT 45 Gy/30f	63				24	3	61	
Bo Qiu (2021)	China	85	HypoTRT 65 Gy/26f	58	24.3	9	Prospective	58	55	63	EP
		92	HyperTRT 45 Gy/30f	58				63	49	67	
Jing Zhang (2017)	China	31	HypoTRT 55 Gy/22 f	59	30	7	Retrospective	8	7	46	EP /EC
		31	Conv TRT 60 Gy/30 f	57				10	12	48	
Joanna Socha (2015)	Poland	100	HypoTRT 42 Gy/15f	59	31	8	Retrospective	24	2	52	EP /EC
		82	Conv TRT 44–60 Gy/22‐30f	59				15	5	37	
Michael Yan (2021)	Canada	63	HypoTRT 40 Gy/15f	68.5	20.4	8	Retrospective			41	Not mentioned
		110	HyperTRT 45 Gy/30f	65.7						80	
Sondos Zayed (2020)	Canada	56	HypoTRT 40 Gy/15f, 45 Gy/15f, 45 Gy/20f	63.3	162	9	Retrospective	51	22	30	Not mentioned
		61	ConvTRT 60 Gy/30f, 66 Gy/33f	68.2	60			51	30	42	
Dan Han (2015)	China	63	HyperTRT 45 Gy/30f	58	27.14	7	Retrospective				EP
		80	ConvTRT 60 Gy//30f	55							
Abhilash Gazula (2014)	America	26	HyperTRT 45 Gy/30f	59	30	7	Retrospective	20	2		EP/EC/CPT‐P/CPT‐C
		19	ConvTRT50‐66.6Gy/25‐37f	65				11	7		
Andrew t. Turrisi (1999)	America	211	HyperTRT 45 Gy/30f	61		7	Prospective	130	38		EP
		206	ConvTRT45 Gy//25f	63				90	39		
By James A. Bonner(1999)	America	130	HyperTRT 45Gy/30f		39	7	Prospective	16	8		EP
		132	ConvTRT50.4 Gy//28f					7	6		
Corinne Faivre‐Finn (2017)	UK	274	HyperTRT 45 Gy/30f	62	45	7	Prospective	206	56		EP
		273	ConvTRT 66 Gy/33f	63				182	55		
John M. Watkins (2009)	America	54	HyperTRT≥HyperTRT 45 Gy/30f	62	26.2	7	Retrospective	11	2		EP
		17	ConvTRT≥59.4G/28f	59				4	1		
Marianna Christodoulou (2018)	Netherlands	29	HyperTRT 45 Gy/30f	73		7	Prospective				EP
		38	ConvTRT 66 Gy/33f								
By Noah C. Choi (1998)	America	24	HyperTRT≥45 Gy/30‐36f	60		7	Prospective	13	0		EP
		19	ConvTRT≥56 Gy/28					3	0		
Natsuo Tomita (2009)	Japan	37	HyperTRT 45 Gy/30f	58		8	Retrospective				EP/EC/CPT‐C
		61	ConvTRT≥54 Gy//27f	66							
		29	ConvTRT<54 Gy/27f	70							
David Schreiber (2015)	America	2821	HyperTRT 45 Gy/30f	65			Retrospective				
		996	ConvTRT 45 Gy/25f								
		11,116	ConvTRT46‐59.4 Gy/23‐33f								
		5095	ConvTRT60‐61.2 Gy/30‐34f								
		5017	ConvTRT62‐72 Gy/31‐40f								
Steven E. Schild (2004)	America	130	HyperTRT48 Gy/32f	62.5	88.8		Prospective	16	11		EP
		131	ConvTRT 50.4 Gy/28f	63				7	6		

### 3.2 Meta‐analysis

#### 3.2.1 Radiotherapy regimen and PFSof LS‐SCLC

**FIGURE 2:**
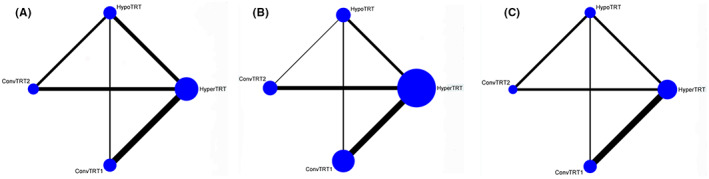
Network of comparisons for (A) PFS, (B) OS, and (C) acute radioactive esophagitis and radioactive pneumonia. Node size represents number of patients in that treatment group. Each line connects two treatment groups those were compared in a study. Line width represents number of trials comparing two treatment groups

**FIGURE 3:**
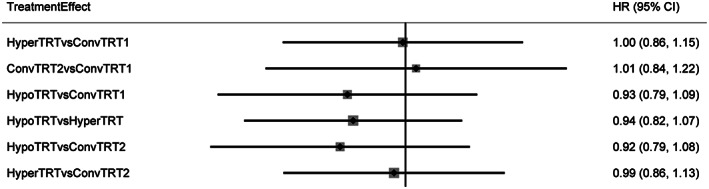
HR of PFS outcomes. HR<1 indicates better outcomes compared to control

Fourteen studies were included in our PFS analysis. The plot of network is shown in Figure 2A. Compared to ConvTRT(1,2), HyperTRT, and HypoTRT seemed to show better for PFS of LS‐SCLC. However, these differences were not statistically significant (HR = 1.00, 95%CI: 0.86–1.15; HR = 0.99, 95%CI: 0.86–1.13; HR = 0.93, 95%CI: 0.79–1.09; HR = 0.92, 95%CI: 0.79–1.08), as seen by the overlapping confidence intervals in Figure 3. HypoTRT were not superior to HyperTRT for PFS of LS‐SCLC(HR = 0.94, 95%CI:0.82–1.07). There was no significant difference between ConvTRT1 and ConvTRT2 for PFS of LS‐SCLC(HR = 1.01, 95%CI:0.84–1.22) (Figure 3).

#### 3.2.2 Radiotherapy regimen and OSof LS‐SCLC

**FIGURE 4:**
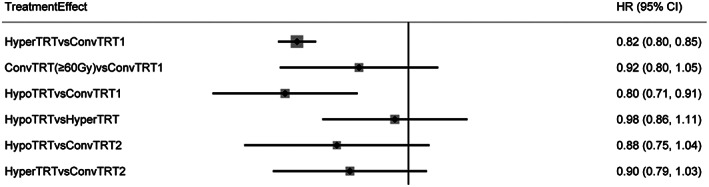
HR of OS outcomes. HR<1 indicates better outcomes compared to control

Nineteen studies reported the effect of radiotherapy on OS of LS‐SCLC. The plot of network is shown in Figure 2B. HyperTRT and HypoTRT significantly improved OS of LS‐SCLC compared to ConvTRT1, with statistical significance(HR = 0.82, 95%CI:0.80–0.85; HR = 0.80, 95%CI:0.71–0.91;). HyperTRT and HypoTRT were beneficial to OS of LS‐SCLC than ConvTRT2, but there was no significant difference (HR = 0.90, 95%CI:0.79–1.03; HR = 0.88, 95%CI:0.75–1.04;). Compared with ConvTRT1, ConvTRT2 did not improve OS of LS‐SCLC (HR = 0.92, 95%CI:0.80–1.05) (Figure 4).

#### 3.2.3 Effect of radiotherapy regimen on acute radioactive esophagitis

**FIGURE 5:**
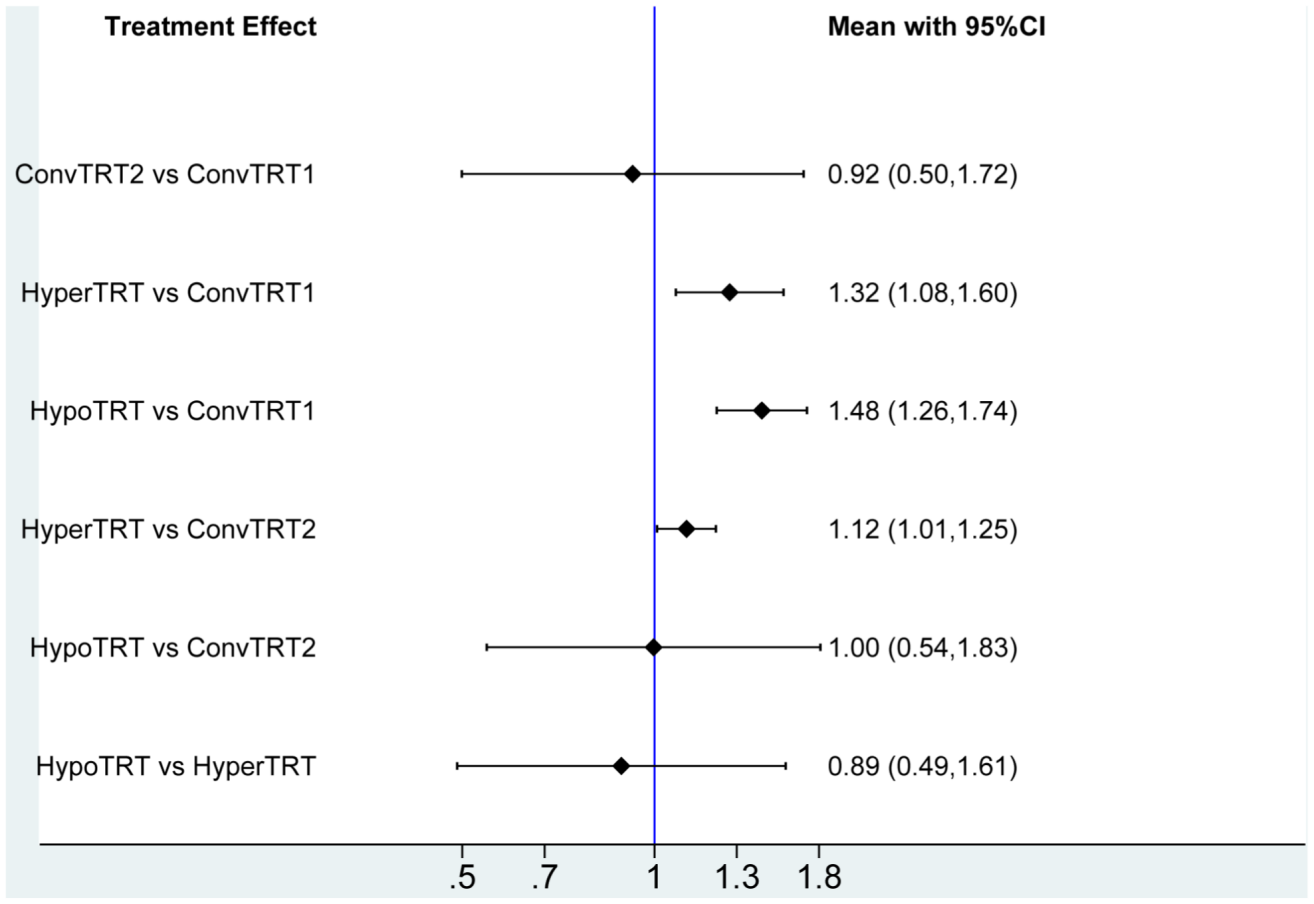
RR of acute radioactive esophagitis outcomes. RR<1 indicates better outcomes compared to control

Acute radioactive esophagitis was reported in 11 studies. The plot of network is shown in Figure 2C. Compared to ConvTRT(1, 2), patients receiving HyperTRT had the highest odds of developing acute radioactive esophagitis with statistical significance (HR = 1.32, 95%CI:1.08–1.60; HR = 1.12, 95%CI: 1.01–1.25). Patients receiving HypoTRT were more likely to develop acute radioactive esophagitis than patients receiving ConvTRT1 (HR = 1.48, 95%CI: 1.26–1.74). Patients receiving HypoTRT were equally likely to develop acute radioactive esophagitis than patients receiving ConvTRT2 (HR = 1.00, 95%CI: 0.54–1.83). There was no significant difference in the incidence of acute radioactive esophagitis between HypoTRT and HyperTRT (HR = 0.89, 95%CI:0.49–1.61), ConvTRT1 and ConvTRT2 (HR = 0.92, 95%CI:0.50–1.72), respectively (Figure 5).

#### 3.2.4 Effect of radiotherapy regimen on acute radioactive pneumonia

**FIGURE 6:**
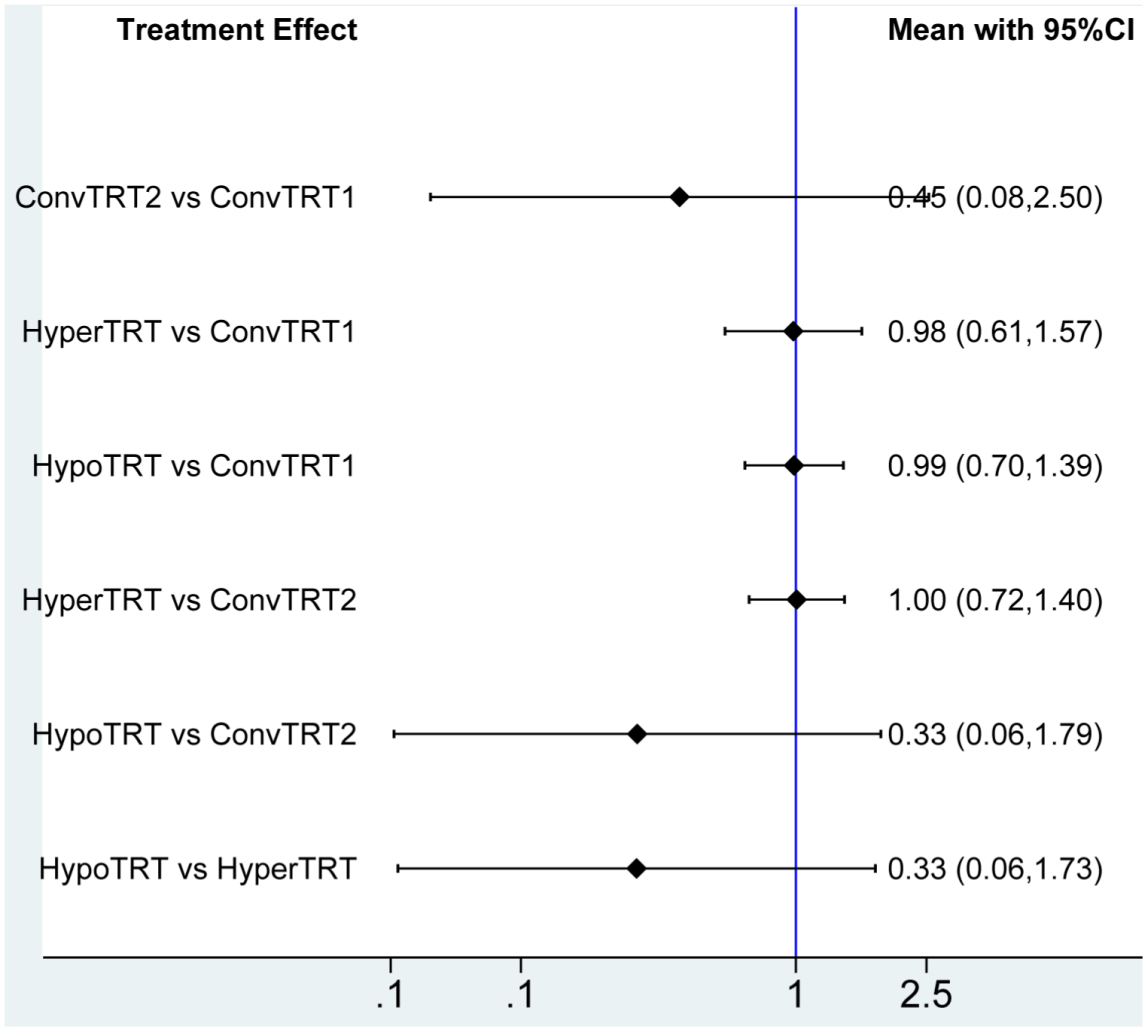
RR of acute radioactive pneumonia outcomes. RR<1 indicates better outcomes compared to control

We included data from 11 studies to evaluate acute radioactive pneumonia. The plot of network is shown in Figure 2C. Patients receiving different radiotherapy regimen did not seemed to have statistically different incidence of acute radioactive pneumonia (HR = 0.45, 95%CI:0.28–2.50; HR = 0.98, 95%CI:0.61–1.57; HR = 0.99, 95%CI:0.70–1.39; HR = 1.00, 95%CI: 0.72–1.40; HR = 0.33, 95%CI: 0.06–1.79; HR = 0.33, 95%CI:0.06–1.73) (Figure 6).

### 3.3 Probability ranking diagram

**FIGURE 7:**
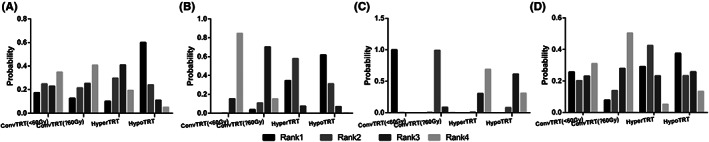
Ranking of four radiotherapy regimens(Rank 1 is best). (A) PFS, (B) OS, (C) acute radioactive esophagitis, (D) acute radioactive pneumonia

The ranking probability map was plotted by calculating the ranking probability of each radiotherapy regimen. The ranking first probability value of ConvTRT1, ConvTRT2, HyperTRT, and HypoTRT for PFS were 0.174, 0.126, 0.100, and 0.600, respectively. For OS, the ranking first probability values of ConvTRT1, ConvTRT2, HyperTRT, and HypoTRT were 0, 0.037, 0.345, and 0.617, respectively. The ranking first probability values for ConvTRT1, ConvTRT2, HyperTRT, and HypoTRT were 0.998, 0.2, 0, and 0, respectively in the incidence of acute radioactive esophagitis. In the incidence of acute radioactive pneumonia, the ranking first probability values for ConvTRT1, ConvTRT2, HyperTRT, and HypoTRT were 0.256, 0.078, 0.291, and 0.375, respectively.(Figure 7).

### 3.4 Consistency test

The loop consistency was analyzed for the four outcomes. The results showed that there were two closed loops in every outcome, without inconsistencies with *P* > 0.05.(Table 2).

**TABLE 2:** Consistency detection

	Loop	IF	Z	P	CI_95
PFS	01–02‐04	0.234	0.471	0.638	(0.00,1.21)
	01–02‐03	0.213	1.072	0.284	(0.00,0.60)
OS	01–02‐04	0.123	0.626	0.531	(0.00,0.51)
	01–02‐03	0.035	0.179	0.858	(0.00,0.42)
Radioactive Esophagitis	01–02‐04	0.107	0.336	0.737	(0.00,0.73)
	01–02‐03	0.028	0.219	0.827	(0.00,0.28)
Radioactive pneumoniti	01–02‐04	1.327	1.196	0.232	(0.00,3.50)
	01–02‐03	0.497	1.775	0.076	(0.00,1.05)

### 3.5 Publication bias

**FIGURE 8:**
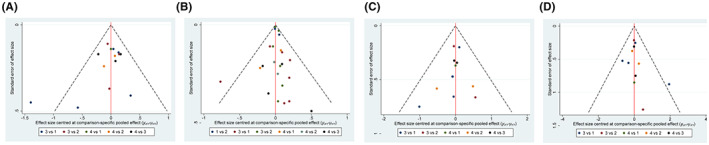
Funnel plots. (A) PFS, (B) OS, (C) acute radioactive esophagitis, (D) acute radioactive pneumonia

The funnel diagram of OS showed a biased distribution, suggesting that there was a certain publication bias. The funnel diagram was relatively symmetrical in PFS, acute radioactive esophagitis, and acute radioactive pneumonia, suggesting that there was less publication bias in the results. In addition, some scattered points were distributed at the bottom of the funnel diagram, suggesting that the results might be affected by the small samples (Figure 8).

## 4 DISCUSSION

It has been reported that the median survival time of LS‐SCLC is 15–20 months, suggesting that the tumor is extremely malignant.^33^ For LS‐SCLC that is not suitable for surgery, the current standard treatment is concurrent chemoradiotherapy.^34^ However, there is much debate about the optimal dose and fractionations of radiotherapy regimen. This was the first network meta‐analysis of the contemporary radiotherapy regimens used in LS‐SCLC that are recommended or not by the 2020 NCCN guidelines to determine the optimal scheme.

Several conclusions could be drawn from the research. First, the contemporary radiotherapy regimens had similar efficacy for PFS of LS‐SCLC and therefore could all be acceptable options for treatment. Second, HypoTRT and HyperTRT significantly improved the OS of LS‐SCLC compared with ConvTRT1(<60 Gy), while not improving the OS of LS‐SCLC compared with ConvTRT2(≥60 Gy). There was no significant difference between HypoTRT and HyperTRT, between ConvTRT1(<60 Gy) and ConvTRT2(≥60 Gy), respectively. Third, HyperTRT developed the highest odds of acute radioactive esophagitis compared to ConvTRT1(<60 Gy) and ConvTRT2(≥60 Gy). HypoTRT were more likely to develop acute radioactive esophagitis than ConvTRT1(<60 Gy). There was no significant difference in the incidence of acute radioactive esophagitis between HypoTRT and HyperTRT, between HypoTRT and ConvTRT2, respectively. Fourth, there was no statistically significant difference among radiotherapy regimens for incidence of acute radioactive pneumonia. Finally, the first ranked treatments in term of longer survival (for PFS and OS) and lower risk for acute radioactive esophagitis and acute radioactive pneumonia were HypoTRT, HypoTRT, ConvTRT1(<60 Gy), and HypoTRT, respectively.

HyperTRT and ConvTRT2(≥60 Gy) have been recommended by the NCCN on the basis of clinical efficacy in separate clinical trials. Our data supported the recommendation made by the NCCN. Our data did not support the results of these previous meta‐analysis which revealed twice‐daily thoracic radiotherapy appeared to be better than once‐daily for LS‐SCLC with better antitumor effects (OS) and similar adverse effects.^35^, ^36^ One caveat to consider was that neither did the two studies strictly distinguish HypoTRT and ConvTRT in once‐daily thoracic radiotherapy group, nor did they consider the effect of total doses in ConvTRT group. In addition, Lin Yang's study did not include a prospective study. On the basis of these results, it was reasonable to assume that HyperTRT might be equivalent ConvTRT2(≥60 Gy). So, HyperTRT and ConvTRT2(≥60 Gy) can be used for LS‐SCLC, which are in line with the NCCN guidelines.

We also found that HypoTRT had similar effectiveness in improving OS compared with HyperTRT and ConvTRT2(≥60 Gy). However, HypoTRT has additional benefits compared with HyperTRT and ConvTRT2. First, HypoTRT can increase the treatment rate, reduce medical expenses, and make patient more comfortable.^37^ According to the research report, the cost is 31.7% lower when low fractionated whole breast irradiation (WBI) compared with traditional fractionated whole breast in the United States.^38^ A study in Asia also showed that the total cost of hypofractionated WBI was reduced by about one third than conventional fractionated WBI.^39^ From a logistical point of view, HypoTRT is easier to manage for both patients and treatment professionals.^32^, ^40^, ^41^ Second, The higher toxicity rates observed in the Turrisi trial have made people persistently hesitate to BID treatment generally adopted. Similar toxicity shown in this network meta‐analysis made HypoTRT possible. Lastly, the start of chemotherapy until the end of radiotherapy(SER)is an important predictor of survival in patients with LS‐SCLC27. Extending ser 1 week can reduce OS by 1.83% in rapidly proliferating tumors such as SCLC.^42^ Prolonging the total duration of radiotherapy will accelerate repopulation of tumor cells, which is unfavorable to treating tumor.^43^, ^44^ HypoTRT may solve some problems about accelerated repopulation caused by prolonged radiotherapy schedules, such as Hyper/ConvTRT.

Acute radioactive esophagitis is the most common complication in patients with LS‐SCLC receiving radiotherapy. Acute radioactive pneumonia is another treatment‐ related complication. Therefore, in addition to efficacy, we compared adverse events such as acute radioactive esophagitis and pneumonia. Our analysis suggested that contemporary regimens might be equivalent for the incidences of acute radioactive esophagitis and pneumonia except for ConvTRT1(<60 Gy). Thus, radiotherapy doctors can focus on the patient's preference and tolerance when deciding the radiotherapy regimen.

One of the strengths of our study was that we compared the contemporary radiotherapy regimens from all of the available studies which provided these data. We also compared efficacy between ConvTRT1(<60 Gy) and ConvTRT2(≥60 Gy), which revealed that ConvTRT1(<60 Gy) was significantly inferior to ConvTRT2(≥60 Gy) in improving OS of LS‐SCLC. Understanding the efficacy of current radiotherapy regimen is of vital importance for radiotherapy doctors' decision‐making. Considering the similar efficacy of these regimens except for ConvTRT1(<60 Gy), it is important to evaluate other aspects of treatment to help determine the optimum regimen. Both the cost of treatment regimen and the convenience of operation should be important factors in decision‐making.

This study had several limitations. First, the studies included in this network meta‐analysis were not all prospective studies, but some retrospective ones. Second, other treatments in the included study might be different. There were differences in chemotherapy regimen, timing of radiotherapy and chemotherapy, treatment after recurrence and so on. Third, most of the included studies did not distinguish between the two recurrence patterns including local recurrence and distant metastasis, but expressed by PFS. Therefore, the difference between the two relapse patterns was not discussed in this study.

## 5 CONCLUSION

Overall, HypoTRT, HyperTRT, and ConvTRT2(≥60 Gy) regimens fared similarly with regard to efficacy and toxicity, thus providing radiotherapy doctors with flexibility in choosing regimens tailored to patient preference and tolerability. Our analysis suggested that HypoTRT was an acceptable option for LS‐SCLC patients today with the benefit of reducing costs and convenient logistics management. Our analysis does not replace the need for comparison with RCT trials.

## CONFLICT OF INTEREST

The authors declare that they have no conflict of interest.

## AUTHOR CONTRIBUTIONS

Jiupeng Zhou and Quanli Dou made contributions to conception and design, publication search, quality evaluation, data collection, statistics, and manuscript writing;.Yongfeng Zhang and Heng Liu made contributions to statistics and editors, and Hui Guo contributed to conception, design, statistics, and editing.

## Data Availability

Data sharing is not applicable to this article as no new data were created or analyzed in this study.

## References

[1] Sung H , Ferlay J , Siegel RL , et al. Global cancer statistics 2020: GLOBOCAN estimates of incidence and mortality worldwide for 36 cancers in 185 countries. CA Cancer J Clin. 2021;71(3):209‐249. 33538338 10.3322/caac.21660

[2] Drapkin BJ , Rudin CM . Advances in small‐cell lung cancer (SCLC) translational research. Cold Spring Harb Perspect Med. 2021;11(4):a038240. 32513672 10.1101/cshperspect.a038240 PMC8015694

[3] Gazdar AF , Bunn PA , Minna JD . Small‐cell lung cancer: what we know, what we need to know and the path forward. Nat Rev Cancer. 2017;17(12):765. 10.1038/nrc.2017.106 29123245

[4] Simone CB 2nd , Bogart JA , Cabrera AR , et al. Radiation therapy for small cell lung cancer: an ASTRO clinical practice guideline. Pract Radiat Oncol. 2020;10(3):158‐173. 32222430 10.1016/j.prro.2020.02.009 PMC10915746

[5] Warde P , Payne D . Does thoracic irradiation improve survival and local control in limited‐stage small‐cell carcinoma of the lung? A meta‐analysis. J Clin Oncol. 1992;10(6):890‐895. 1316951 10.1200/JCO.1992.10.6.890

[6] De Ruysscher D , Lueza B , Le Péchoux C , et al. Impact of thoracic radiotherapy timing in limited‐stage small‐cell lung cancer: usefulness of the individual patient data meta‐analysis. Ann Oncol. 2016;27(10):1818‐1828. 27436850 10.1093/annonc/mdw263 PMC5035783

[7] Faivre‐Finn C , Snee M , Ashcroft L , et al. Concurrent once‐daily versus twice‐daily chemoradiotherapy in patients with limited‐stage small‐cell lung cancer (CONVERT): an open‐label, phase 3, randomised, superiority trial. Lancet Oncol. 2017;18(8):1116‐1125. 28642008 10.1016/S1470-2045(17)30318-2 PMC5555437

[8] Choi NC , Herndon JE 2nd , Rosenman J , et al. Phase I study to determine the maximum‐tolerated dose of radiation in standard daily and hyperfractionated‐accelerated twice‐daily radiation schedules with concurrent chemotherapy for limited‐stage small‐cell lung cancer. J Clin Oncol. 1998;16(11):3528‐3536. 9817271 10.1200/JCO.1998.16.11.3528

[9] Miller KL , Marks LB , Sibley GS , et al. Routine use of approximately 60 Gy once‐daily thoracic irradiation for patients with limited‐stage small‐cell lung cancer. Int J Radiat Oncol Biol Phys. 2003;56(2):355‐359. 12738309 10.1016/s0360-3016(02)04493-0

[10] Roof KS , Fidias P , Lynch TJ , Ancukiewicz M , Choi NC . Radiation dose escalation in limited‐stage small‐cell lung cancer. Int J Radiat Oncol Biol Phys. 2003;57(3):701‐708. 14529774 10.1016/s0360-3016(03)00715-6

[11] Bogart JA , Herndon JE 2nd , Lyss AP , et al. 70 Gy thoracic radiotherapy is feasible concurrent with chemotherapy for limited‐stage small‐cell lung cancer: analysis of cancer and leukemia group B study 39808. Int J Radiat Oncol Biol Phys. 2004;59(2):460‐468. 15145163 10.1016/j.ijrobp.2003.10.021

[12] Turrisi AT 3rd , Kim K , Blum R , et al. Twice‐daily compared with once‐daily thoracic radiotherapy in limited small‐cell lung cancer treated concurrently with cisplatin and etoposide. N Engl J Med. 1999;340(4):265‐271. 9920950 10.1056/NEJM199901283400403

[13] Grant JD , Shirvani SM , Tang C , et al. Incidence and predictors of severe acute esophagitis and subsequent esophageal stricture in patients treated with accelerated hyperfractionated chemoradiation for limited‐stage small cell lung cancer. Pract Radiat Oncol. 2015;5(4):e383‐e391. 25731965 10.1016/j.prro.2015.01.005

[14] Grønberg BH , Halvorsen TO , Fløtten Ø , et al. Randomized phase II trial comparing twice daily hyperfractionated with once daily hypofractionated thoracic radiotherapy in limited disease small cell lung cancer. Acta Oncol. 2016;55(5):591‐597. 26494411 10.3109/0284186X.2015.1092584

[15] Qiu B , Li Q , Liu J , et al. Moderately Hypofractionated once‐daily compared with twice‐daily thoracic radiation therapy concurrently with etoposide and cisplatin in limited‐stage small cell lung cancer: a multicenter, phase II, randomized trial. Int J Radiat Oncol Biol Phys. 2021;111(2):424‐435. 33992717 10.1016/j.ijrobp.2021.05.003

[16] Li T , Puhan MA , Vedula SS , Singh S , Dickersin K . Network meta‐analysis‐highly attractive but more methodological research is needed. BMC Med. 2011;9:79. 21707969 10.1186/1741-7015-9-79 PMC3159133

[17] Tierney JF , Stewart LA , Ghersi D , Burdett S , Sydes MR . Practical methods for incorporating summary time‐to‐event data into meta‐analysis. Trials. 2007;8:16. 17555582 10.1186/1745-6215-8-16 PMC1920534

[18] Parmar MK , Torri V , Stewart L . Extracting summary statistics to perform meta‐analyses of the published literature for survival endpoints. Stat Med. 1998;17(24):2815‐2834. 9921604 10.1002/(sici)1097-0258(19981230)17:24<2815::aid-sim110>3.0.co;2-8

[19] Bonner JA , Sloan JA , Shanahan TG , et al. Phase III comparison of twice‐daily split‐course irradiation versus once‐daily irradiation for patients with limited stage small‐cell lung carcinoma. J Clin Oncol. 1999;17(9):2681‐2691. 10561342 10.1200/JCO.1999.17.9.2681

[20] Christodoulou M , Blackhall F , Mistry H , et al. Compliance and outcome of elderly patients treated in the concurrent once‐daily versus twice‐daily radiotherapy (CONVERT) trial. J Thorac Oncol. 2019;14(1):63‐71. 30391573 10.1016/j.jtho.2018.09.027 PMC6328625

[21] Schild SE , Bonner JA , Shanahan TG , et al. Long‐term results of a phase III trial comparing once‐daily radiotherapy with twice‐daily radiotherapy in limited‐stage small‐cell lung cancer. Int J Radiat Oncol Biol Phys. 2004;59(4):943‐951. 15234027 10.1016/j.ijrobp.2004.01.055

[22] Videtic GM , Truong PT , Dar AR , Yu EW , Stitt LW . Shifting from hypofractionated to "conventionally" fractionated thoracic radiotherapy: a single institution's 10‐year experience in the management of limited‐stage small‐cell lung cancer using concurrent chemoradiation. Int J Radiat Oncol Biol Phys. 2003;57(3):709‐716. 14529775 10.1016/s0360-3016(03)00635-7

[23] Bettington CS , Tripcony L , Bryant G , Hickey B , Pratt G , Fay M . A retrospective analysis of survival outcomes for two different radiotherapy fractionation schedules given in the same overall time for limited stage small cell lung cancer. J Med Imaging Radiat Oncol. 2013;57(1):105‐112. 23374562 10.1111/j.1754-9485.2012.02470.x

[24] Zhang J , Fan M , Liu D , et al. Hypo‐ or conventionally fractionated radiotherapy combined with chemotherapy in patients with limited stage small cell lung cancer. Radiat Oncol. 2017;12(1):51. 28283034 10.1186/s13014-017-0788-x PMC5346226

[25] Socha J , Guzowska A , Tyc‐Szczepaniak D , et al. Accelerated hypofractionated thoracic radiotherapy in limited disease small cell lung cancer: comparison with the results of conventionally fractionated radiotherapy. J Buon. 2015;20:146‐157. 25778310

[26] Yan M , Sigurdson S , Greifer N , et al. A comparison of Hypofractionated and twice‐daily thoracic irradiation in limited‐stage small‐cell lung cancer: an overlap‐weighted analysis. Cancers (Basel). 2021;13(12):2895. 34207857 10.3390/cancers13122895 PMC8229231

[27] Zayed S , Chen H , Ali E , et al. Is there a role for Hypofractionated thoracic radiation therapy in limited‐stage small cell lung cancer? A propensity score matched analysis. International Journal of Radiation Oncology*Biology*Physics. 2020;108(3):575‐586. 32544575 10.1016/j.ijrobp.2020.06.008 PMC7293491

[28] Han D , Hao S , Tao C , et al. Comparison of once daily radiotherapy to 60 Gy and twice daily radiotherapy to 45 Gy for limited stage small‐cell lung cancer. Thorac Cancer. 2015;6(5):643‐648. 26445614 10.1111/1759-7714.12262 PMC4567011

[29] Gazula A , Baldini EH , Chen A , Kozono D . Comparison of once and twice daily radiotherapy for limited stage small‐cell lung cancer. Lung. 2014;192(1):151‐158. 24162870 10.1007/s00408-013-9518-9

[30] Watkins JM , Fortney JA , Wahlquist AE , et al. 3rd, Sharma AK: once‐daily radiotherapy to > or =59.4 Gy versus twice‐daily radiotherapy to > or =45.0 Gy with concurrent chemotherapy for limited‐stage small‐cell lung cancer: a comparative analysis of toxicities and outcomes. Jpn J Radiol. 2010;28(5):340‐348. 20585921 10.1007/s11604-010-0429-x

[31] Tomita N , Kodaira T , Hida T , et al. The impact of radiation dose and fractionation on outcomes for limited‐stage small‐cell lung cancer. Int J Radiat Oncol Biol Phys. 2010;76(4):1121‐1126. 19665321 10.1016/j.ijrobp.2009.03.069

[32] Schreiber D , Wong AT , Schwartz D , Rineer J . Utilization of Hyperfractionated radiation in small‐cell lung cancer and its impact on survival. J Thorac Oncol. 2015;10(12):1770‐1775. 26334750 10.1097/JTO.0000000000000672

[33] Sun A , Durocher‐Allen LD , Ellis PM , et al. Guideline for the initial Management of Small Cell Lung Cancer (limited and extensive stage) and the role of thoracic radiotherapy and first‐line chemotherapy. Clin Oncol (R Coll Radiol). 2018;30(10):658‐666. 30007803 10.1016/j.clon.2018.06.008

[34] Blackhall F , Frese KK , Simpson K , Kilgour E , Brady G , Dive C . Will liquid biopsies improve outcomes for patients with small‐cell lung cancer? Lancet Oncol. 2018;19(9):e470‐e481. 30191851 10.1016/S1470-2045(18)30455-8

[35] Wu Q , Xiong Y , Zhang S , et al. A meta‐analysis of the efficacy and toxicity of twice‐daily vs. once‐daily concurrent chemoradiotherapy for limited‐stage small cell lung cancer based on randomized controlled trials. Front Oncol. 2019;9:1460. 31970086 10.3389/fonc.2019.01460 PMC6960125

[36] Yang L, Liu L, Yang Y, Lei Y , Wang T , Wu X , Guo X : Twice‐daily vs higher‐dose once‐daily thoracic radiotherapy for limited‐disease small‐cell lung cancer: a PRISMA‐compliant meta‐analysis. Medicine (Baltimore) 2020, 99(27):e20518. 32629632 10.1097/MD.0000000000020518 PMC7337461

[37] Zietman AL : Making radiation therapy for prostate cancer more economical and more convenient. J Clin Oncol. 2016, 34(20):2323–2324. 27091711 10.1200/JCO.2016.67.3764

[38] Greenup RA , Camp MS , Taghian AG , et al. Cost comparison of radiation treatment options after lumpectomy for breast cancer. Ann Surg Oncol. 2012;19(10):3275‐3281. 22851048 10.1245/s10434-012-2546-5

[39] Karasawa K , Kunogi H , Hirai T , et al. Comparison of hypofractionated and conventionally fractionated whole‐breast irradiation for early breast cancer patients: a single‐institute study of 1,098 patients. Breast Cancer. 2014;21(4):402‐408. 22968629 10.1007/s12282-012-0406-6

[40] Glatzer M , Faivre‐Finn C , De Ruysscher D , et al. Once daily versus twice‐daily radiotherapy in the management of limited disease small cell lung cancer ‐ decision criteria in routine practise. Radiother Oncol. 2020;150:26‐29. 32447035 10.1016/j.radonc.2020.05.004

[41] Woolf DK , Slotman BJ , Faivre‐Finn C . The current role of radiotherapy in the treatment of small cell lung cancer. Clin Oncol (R Coll Radiol). 2016;28(11):712‐719. 27522475 10.1016/j.clon.2016.07.012

[42] De Ruysscher D , Pijls‐Johannesma M , Bentzen SM , et al. Time between the first day of chemotherapy and the last day of chest radiation is the most important predictor of survival in limited‐disease small‐cell lung cancer. J Clin Oncol. 2006;24(7):1057‐1063. 16505424 10.1200/JCO.2005.02.9793

[43] Lee KJ, Lee EJ, Hur GY , Lee SY , Kim JH , Shin C , Shim JJ , In KH , Kang KH , Yoo SH *et al*: The start of chemotherapy until the end of radiotherapy in patients with limited‐stage small cell lung cancer. Korean J Intern Med 2013, 28(4):449–455. 23864803 10.3904/kjim.2013.28.4.449 PMC3712153

[44] Brade AM , Tannock IF . Scheduling of radiation and chemotherapy for limited‐stage small‐cell lung cancer: repopulation as a cause of treatment failure? J Clin Oncol. 2006;24(7):1020‐1022. 16505418 10.1200/JCO.2005.04.9676

